# Chlorogenic Acid Enhances Beta‐Lapachone‐Induced Cell Death by Suppressing Autophagy in NQO1‐Positive Cancer Cells

**DOI:** 10.1002/cbin.70006

**Published:** 2025-02-27

**Authors:** Sahib Zada, Md Entaz Bahar, Wanil Kim, Deok Ryong Kim

**Affiliations:** ^1^ Department of Biochemistry and Convergence Medical Science, Institute of Medical Science Gyeongsang National University College of Medicine JinJu Republic of Korea

**Keywords:** apoptosis, autophagy, beta‐Lapachone, chlorogenic acid, combination therapy, PKA

## Abstract

Resistance to apoptosis‐inducing drugs frequently occurs in cancer cells, limiting their usefulness in ongoing cancer treatment. Despite ongoing efforts to overcome drug resistance, a definitive solution remains elusive. However, autophagy inhibition has been shown to enhance the effectiveness of some anticancer drugs and is a possible strategy for overcoming drug resistance. In this study, we demonstrate that chlorogenic acid (CGA), a natural antioxidant, significantly enhances beta‐lapachone (β‐Lap)‐induced cell death in cancer cells. The augmented apoptosis induced by CGA is associated with activation of protein kinase A (PKA) in β‐Lap–treated cells, independent of the antioxidant properties of CGA. As a result, PKA activation in cancer cells co‐treated with β‐Lap and CGA effectively inhibits autophagy. Notably, PKA activation leads to phosphorylation of microtubule‐associated protein 1 A/1B‐light chain 3 (LC3) at the serine 12 residue, causing autophagy suppression irrespective of mTORC activity. Importantly, the cell death induced by β‐Lap and CGA in NQO1‐overexpressing breast or lung cancers is closely linked to autophagy inhibition. These findings suggest that combining β‐Lap and CGA might be a novel strategy for cancer therapy, particularly for overcoming drug resistance caused by autophagy induction in cancer cells.

**Abbreviations**
AO: acridine orange
CGA: chlorogenic acid
CQ: chloroquine
DCFDA: 2′,7′‐dichloroflourescin diacetate
LC3: microtubule‐associated protein 1A/1B‐light chain 3
MDC: monodansylcadaverine
mTOR1: mammalian target of rapamycin complex 1
NAC: N‐acetylcysteine
NQO1: NAD(P)H oxidoreductase 1
PARP1: poly(ADP‐ribose) polymer 1
PKA: protein kinase A
ROS: reactive oxygen species
β‐Lap: beta‐Lapachone

## 1 Introduction

Over the past few decades, combination cancer therapy has primarily focused on enhancing the likelihood and magnitude of therapeutic responses while minimizing the potential for drug resistance. Early clinical studies demonstrated the feasibility of successive combination regimens, particularly for patients displaying varying responses to drugs due to tumor heterogeneity (Palmer et al. 2019, Pritchard et al. 2013). Targeted therapies are often combined based on their specific molecular mechanisms, as well as evidence supporting their additive or synergistic effects in cancer cells (Kummar et al. 2010). For instance, in patients with blood cancer, survival rates can reach as high as 80% when multiple agents are employed, whereas monotherapy with a single drug often yields poor results (Pritchard et al. 2013). Furthermore, various strategies involving combinations of multiple drugs have been explored to enhance clinical management and improve the survival of patients with non‐small cell lung cancer (Sun et al. 2021). The natural compound β‐lapachone (β‐Lap) exhibits diverse physiological roles in numerous cancer types, including breast and lung cancers (Bey et al. 2007, Li et al. 2011). Its versatile effects on cancer have led to its utilization in several clinical trials with cancer patients (Gerber et al. 2018, Miao et al. 2009). The natural antioxidant chlorogenic acid (CGA) also plays an important role in the functions of various cells (Farah et al. 2008). CGA serves as a chemo‐sensitizing reagent, effectively suppressing cancer growth, and can activate or inhibit specific metabolic pathways within cancer cells (Lukitasari et al. 2018).

β‐Lap exhibits synergistic potential as an anticancer drug when used in combination with radiotherapy (Beg et al. 2017) and various chemicals, including taxols, DNA‐damaging reagents, and gemcitabine (Arnrich and Freitag 1983, D'Anneo et al. 2010, Li et al. 1999). These combination therapies induce sufficient DNA damage to trigger hyperactivation of PARP1 and subsequent cell death, even at sublethal doses of the individual agents (Dong et al. 2010, Huang et al. 2016, Kim et al. 2005). CGA augments the anticancer effect of other drugs by promoting overproduction of intracellular reactive oxygen species (ROS) and subsequent inhibition of cell proliferation in multiple cancer types (Catanzaro et al. 2016, Yan et al. 2015). Notably, β‐Lap elevates ROS levels by activating NQO1 in cancer cells (Lamberti et al. 2013, Li et al. 2014). Therefore, combination therapy involving CGA and β‐Lap may be an optimal approach to amplify the anticancer effects of β‐Lap through CGA. In our previous study, β‐Lap‐induced cell death through protein kinase A (PKA) activation and autophagy modulation, although the precise mechanisms remain unclear (Zada et al. 2019a). PKA can play either a proapoptotic or an antiapoptotic role in cancer (Hedrick et al. 2013, Naviglio et al. 2009). Moreover, PKA regulates cell death pathways through various mechanisms, involving both autophagy and apoptosis, in many cancer cells (Budovskaya et al. 2004, Holen et al. 1996). Autophagy is a cellular response to various forms of intracellular damage or extracellular stressors, including protein aggregation, damaged organelles, and deprivation of nutrients or growth factors crucial for cell survival during cancer treatment (Amaravadi et al. 2016, Haas et al. 2019). Rapamycin, an mTOR inhibitor, activates autophagy and is widely investigated for its anticancer effects; however, its clinical use is constrained because of the differing responses it elicits in cancer cells (Bahar et al. 2023, Holloway and Marignani 2021, Mukhopadhyay et al. 2016, Stephan et al. 2009). In this context, autophagy inhibition can enhance the effectiveness of anticancer drugs and has shown potential in overcoming resistance to chemotherapy (Dong et al. 2021). Indeed, autophagic inhibitors like chloroquine (CQ) and hydroxychloroquine (HCQ) have been found to alleviate chemoresistance in cancer cells and stimulate cell death, offering promising results in combating resistance to anticancer drugs (Fukuda et al. 2015, Galluzzi et al. 2015).

In this study, we demonstrate that β‐Lap‐induced apoptosis in cancer cells can be enhanced by the addition of CGA. This enhancement of cell death by CGA in β‐Lap–treated cells is not linked to the antioxidant and ROS properties of CGA. Instead, co‐treatment with β‐Lap and CGA notably activates apoptosis in a PKA‐dependent manner. PKA activation results in phosphorylation of microtubule‐associated protein 1A/1B‐light chain 3 (LC3) at the serine 12 residue, leading to inhibition of autophagy, which has a substantial impact on cell death induced by β‐Lap in breast and lung cancer cells overexpressing NQO1. These findings suggest that the combination of CGA and β‐Lap might be a novel strategy for the development of new anticancer therapies.

## 2 Materials and Methods

### 2.1 Reagents

Roswell Park Memorial Institute 1640 medium (RPMI‐1640), fetal bovine serum (FBS), and Dulbecco's modified Eagle medium (DMEM; #11995‐065) were purchased from Gibco Life Technologies (Gaithersburg, MD, USA). Acridine orange (AO; #318337), 2′,7′‐dichlorofluorescein diacetate (DCFDA; #D6883), CQ (#C6628), monodansylcadaverine (MDC; #30432), CGA (#SLBJ3632V), β‐Lap (#L2037), N‐acetylcysteine (NAC; #A9165), forskolin (#F3917), dibutyryl‐cAMP (#D0260), and H‐89 (#B1427) were purchased from Sigma‐Aldrich (St. Louis, MO, USA). Rapamycin (#R‐5000) was purchased from LC Laboratories (Woburn, MA, USA). CCK‐8 (Cell Counting Kit‐8, #CK04) was purchased from Dojindo (Tokyo, Japan). The DeadEnd™ Fluorometric TUNEL system (#G3250, lot #0000007545) was purchased from Promega (Madison, WI, USA). Diamond antifade mountant with DAPI (#p36966) was purchased from Invitrogen (Carlsbad, CA, USA). Primary antibodies against caspase‐3 (#9662), cleaved caspase‐3 (#9661), PARP (#9542), cleaved PARP (#9541), MAP1LC3β (#SC‐376404), SQSTM1/p62 (#SC‐28359), MTOR (#2983), and phospho‐MTOR (Ser2448; #2971) were purchased from Cell Signaling Technology (Beverly, MA, USA). Primary antibodies against Bcl‐2 (#7382), Bcl‐X_L_ (#56021), cytochrome C (#13560), Bak (#832), p‐PKAα/β/γ T198 (#32968), and PKAα (#903) were purchased from Santa Cruz Biotechnology (Santa Cruz, CA, USA). Anti‐pLC3 (Ser12) antibody (# LS‐C540497) was purchased from LS Bio (Lynnwood, WA, USA). Antibody against β‐actin (#A5441) was purchased from Sigma‐Aldrich. Secondary antibodies against rabbit, mouse, and goat were purchased from Bio‐Rad (Hercules, CA, USA).

### 2.2 Cell Culture

Two breast cancer cell lines, MDA‐MB‐231 overexpressing NQO1 (231‐NQO1^+/+^) and MDA‐MB‐231 lacking NQO1 (231‐NQO1^−/−^), were obtained from Dr. Boothman (UT Southwestern, USA) as described previously and cultured in RPMI medium supplemented with 5% (v/v) FBS, 100 units/mL penicillin, and 100 µg/mL streptomycin at 37°C in a 5% CO_2_ humidified atmosphere. A549 cells, purchased from the Korean Cell Line Bank (#10185), were cultured in DMEM supplemented with 10% FBS, 100 units/mL penicillin, and 100 µg/mL streptomycin at 37°C in a 5% CO_2_ humidified atmosphere.

### 2.3 Cell Viability

The viability of cells was determined by CCK‐8 analysis as described previously. In brief, 231‐NQO1^+/+^, 231‐NQO1^−/−^, or A549 cells were treated with β‐Lap alone or in combination with CGA in the presence or absence of other inhibitors or activators for 2 h in a 96‐well plate. After removal of the medium, the cells were further incubated in fresh medium for 4 h. After addition of CCK‐8 reagent (10 µL) into each well, the cells were incubated for 4 h at 37°C in a 5% CO_2_ humidified atmosphere. Absorbance was measured at 485 nm using a microplate reader (Hidex 1 FN/Chameleon; Turku, Finland).

### 2.4 Determination of Intracellular ROS

Intracellular ROS contents were determined using a DCFDA ROS detection assay kit (ab113851; Abcam, Burlingame, CA, USA) as described previously. Briefly, 231‐NQO1^+/+^, 231‐NQO1^−/−^, or A549 cells (~5 × 10^4^ cells/well) were seeded into a 96‐well plate containing 200 µL appropriate media (RPMI or DMEM). After incubation for 24 h at 37°C, the cells were treated with β‐Lap alone or in combination with CGA in the presence or absence of inhibitors or activators for 2 h. Then, DCFDA dissolved in DMSO/PBS was added to each well (30 µM final concentration), followed by incubation for 30 min under light‐free conditions. Fluorescence was determined at 485/535 nm using the GloMax® ‐Multiple detection system (Model # E 8032; Promega, Sunnyvale, CA, USA).

### 2.5 Western Blot Analysis

Cells were collected and washed twice with 1× ice‐cold PBS. Total proteins were extracted with cell lysis buffer (#87788; Pierce Biotechnology, Waltham, MA, USA) containing protease and phosphatase inhibitor cocktail (100× Halt™ Proteases & phosphatase single‐use inhibitor cocktail; ThermoScientific), and the protein concentration was determined using a Pierce protein assay kit. Total protein lysates (30 µg) were separated by 10% SDS‐PAGE, and the target proteins were specifically detected by Western blot using the indicated antibodies as described previously. Proteins were visualized with ECL Detection Reagent (ThermoScientific, Scotts Valley, CA, USA) and quantified using ImageJ software (National Institutes of Health, Bethesda, MD, USA). Protein levels were normalized to β‐actin.

### 2.6 Colony Formation Assay

Cells (231‐NQO1^+/+^, 231‐NQO1^−/−^, or A549) were seeded in a 12‐well plate at a density of 750 cells/mL in complete medium. After 24 h, the cells were treated with the indicated drugs for 4 h and then further incubated for 7 days in fresh medium for colony formation. The cells were then gently washed with 1× PBS and fixed in 500 μL 100% ice‐cold methanol at −20°C for 15–20 min. After additional washing with 1× PBS twice, the cells were stained with 500 μL 0.01% crystal violet blue for 10–20 min and washed twice with 1× PBS. After air drying the plate, cell images were captured using a digital camera.

### 2.7 TUNEL Assay

Cells were cultured on coverslips for 24 h and then treated with β‐Lap alone or in combination with CGA in the presence or absence of inhibitors or activators (forskolin, H89, CQ, and rapamycin). The cells were then fixed in 4% (*w*/*v*) paraformaldehyde for 30 min and permeabilized in PBS containing 0.1% Triton X‐100 for 20 min at room temperature. The cells were blocked with 5% horse serum in PBS for 1 h. After washing with PBS, the cells were mounted onto a glass slide with mounting medium containing DAPI. Fluorometric TUNEL analyses (about 1500 nuclei per field) were performed under a fluorescence microscope (BX51‐DSU; Olympus, Tokyo, Japan) as described previously (Lee et al. 2020). The experiments were repeated at least three times.

### 2.8 Immunofluorescence Staining

Staining analysis was performed as described previously. In brief, cells were cultured on a coverslip for 24 h and then treated with 500 mM H_2_O_2_ in the presence of 250 µM CGA for 2 h. The cells were then fixed in 4% (*w*/*v*) paraformaldehyde for 30 min and permeabilized in PBS containing 0.1% Triton X‐100 for 20 min at room temperature. The cells were blocked in PBS with 5% horse serum for 1 h and then incubated with primary antibodies overnight at 4°C. After washing with PBS, the cells were incubated with FITC‐conjugated secondary antibodies (1:50 in PBS) at room temperature for 90 min. After the slides were washed twice with PBS for 5 min, images were captured under a confocal microscope (FV‐1000; Olympus, Tokyo, Japan).

### 2.9 AO Staining

Cells (231‐NQO1^+/+^, 231‐NQO1^−/−^, or A549) were cultured on a coverslip for 24 h and then treated with β‐Lap or/and CGA in the presence of inhibitors or activators for 2 h. Then, the cells were washed three times with 1× PBS, fixed in 4% formaldehyde, and permeabilized in 100% methanol. AO solution was added to the cells at a final concentration of 1 μg/mL and incubated for 15 min at room temperature as described previously. Fluorescence images of acidic vacuoles (red) and cytoplasm (green) were captured under a confocal microscope (FV‐1000; Olympus) using filters for TRITC and FITC, respectively. Overlapping images were then processed and quantified using ImageJ software.

### 2.10 MDC Staining of Autophagic Vacuoles

Cells (231‐NQO1^+/+^, 231‐NQO1^−/−^, or A549) were incubated on a coverslip for 24 h and then treated with β‐Lap or/and CGA in the presence of inhibitors or activators for 2 h. Following incubation, the cells were washed three times with 1× PBS. Autophagic vacuoles were stained by 0.05 mM MDC in PBS at 37°C for 15 min under light‐free conditions. Then, the cells were washed four times with 1× PBS and immediately analyzed under an inverted fluorescence microscope (Leica, Wetzlar, Germany) as described previously.

### 2.11 Determination of Synergy Quotient

The synergism quotient (SQ) was calculated by subtracting baseline values from all treatments, followed by dividing the effects of combined treatments by the sum of individual treatments. An SQ value greater than 1.0 represents synergism for a given response.

### 2.12 Statistical Analysis

Each experiment was conducted independently at least three times, and values are expressed as the mean ± standard deviation (SD). Differences between two groups were assessed by two‐tailed Student's *t*‐test. One‐way analysis of variance was used to compare the means of three or more groups, followed by multiple‐comparison Tukey tests. Values of *p < 0.05 and **p < 0.01 are considered significant.

## 3 Results

### 3.1 CGA Promotes β‐Lap‐Induced Cell Death in NQO1‐Overexpressing Cancer Cells

**Figure 1:**
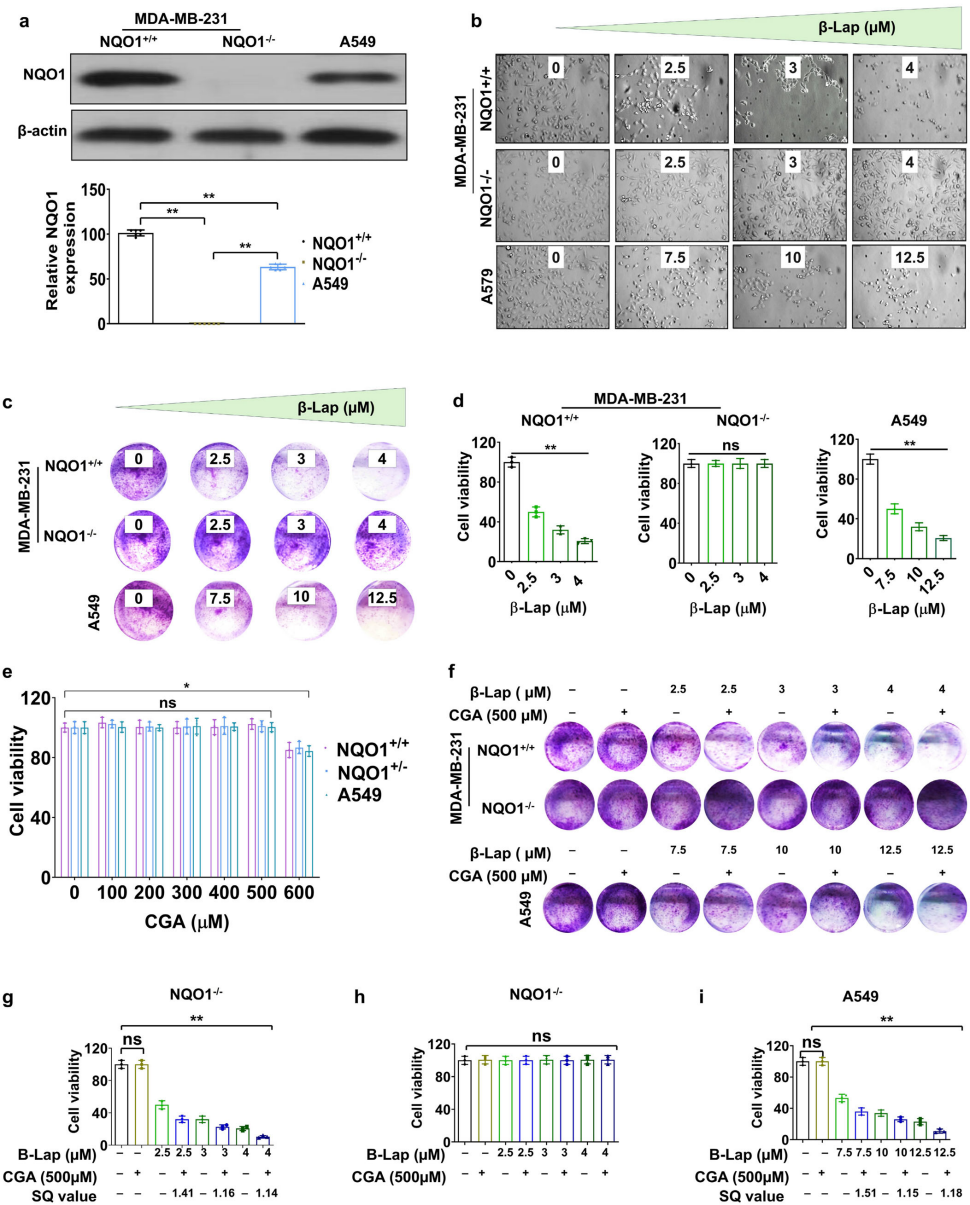
β‐Lap‐induced cell death and its enhancement by CGA in cancer cells that overexpress NQO1. (a) The expression levels of NQO1 proteins in 231‐NQO1^+/+^, 231‐NQO1^−/−^, or A549 cells were determined by western blot and quantified. (b–d) Different concentrations of β‐Lap were administered to 231‐NQO1^+/+^and 231‐NQO1^−/−^ breast cancer cells (0, 2.5, 3, and 4 µM) and A459 lung cancer cells (0, 7.5, 10, and 12.5 µM) for 2 h. After further incubation in fresh medium with 5% FBS for 4 h, the viability and proliferation of cells in each condition were evaluated by cell imaging (b), colony formation assay (c), and CCK‐8 assay (d). Cell viability data represent the mean (±SD) of three independent experiments (**p < 0.01). Colony formation by cancer cells was determined as described in Section 2. All cell images were captured by a bright‐field microscope (100× magnification) or a digital camera. Bar, 10 µm. (e) 231‐NQO1^+/+^, 231‐NQO1^−/−^, or A549 cells were treated with CGA (0, 100, 200, 300, 400, 500, and 600 µM), and their viability was determined by CCK‐8 assay. Data represent the mean (±SD) of three independent experiments (*p < 0.05). *ns* indicates no significance. (f–i) Different concentrations of β‐Lap were administered to 231‐NQO1^+/+^ or 231‐NQO1^−/−^ cells (0, 2.5, 3, and 4 µM) or A459 cells (0, 7.5, 10, and 12.5 µM), each in combination with 500 µM CGA, for 2 h. The cells were subsequently cultured for 4 h in fresh medium (either RPMI or DMEM) with 5% FBS. The viability and proliferation of cells under each condition were determined by colony formation analysis (f) and CCK‐8 assay (g–i), respectively. SQ indicates synergism quotient values. Data represent the mean (±SD) of three independent experiments (**p < 0.01). Cell images were captured by a digital camera. CCK‐8, Cell Counting Kit‐8; CGA, chlorogenic acid; DMEM, Dulbecco's modified Eagle medium; FBS, fetal bovine serum; RPMI‐1640, Roswell Park Memorial Institute 1640 medium; SD, standard deviation.

The anticancer drug β‐Lap exerts its effects selectively on cancer cells that express high levels of NQO1, as these cancer cells generate elevated levels of toxic ROS through a futile cycle reaction involving β‐Lap and NQO1 (Bentle, Bey, et al. 2006, Bentle, Reinicke, et al. 2006, Bentle et al. 2007, Huang et al. 2012, Kim et al. 2014). We initially examined the impact of β‐Lap on cell viability in two syngeneic breast cancer cell lines, 231‐NQO1^+/+^ and 231‐NQO1^−/−^, along with A549 lung cancer cells. As expected, NQO1 was highly expressed in 231‐NQO1^+/+^ cells and A459 cells but not in 231‐NQO1^−/−^ cells (Figure 1a). Next, we treated these cells with various concentrations of β‐Lap (0, 2.5, 3, and 4 µM for 231‐NQO1^+/+^ and 231‐NQO1^−/−^ cells; 0, 7.5, 10, and 12.5 µM for A549 cells) and assessed their viability by CCK8 assay, colony formation assay, and imaging analyses. Cells expressing high levels of NQO1 (231‐NQO1^+/+^ cells and A549 cells) displayed a dose‐dependent decrease in viability with β‐Lap treatment, whereas 231‐NQO1^−/−^ cells exhibited no change in viability, regardless of the β‐Lap dose, across all assays (Figure 1b–d).

**Figure 2:**
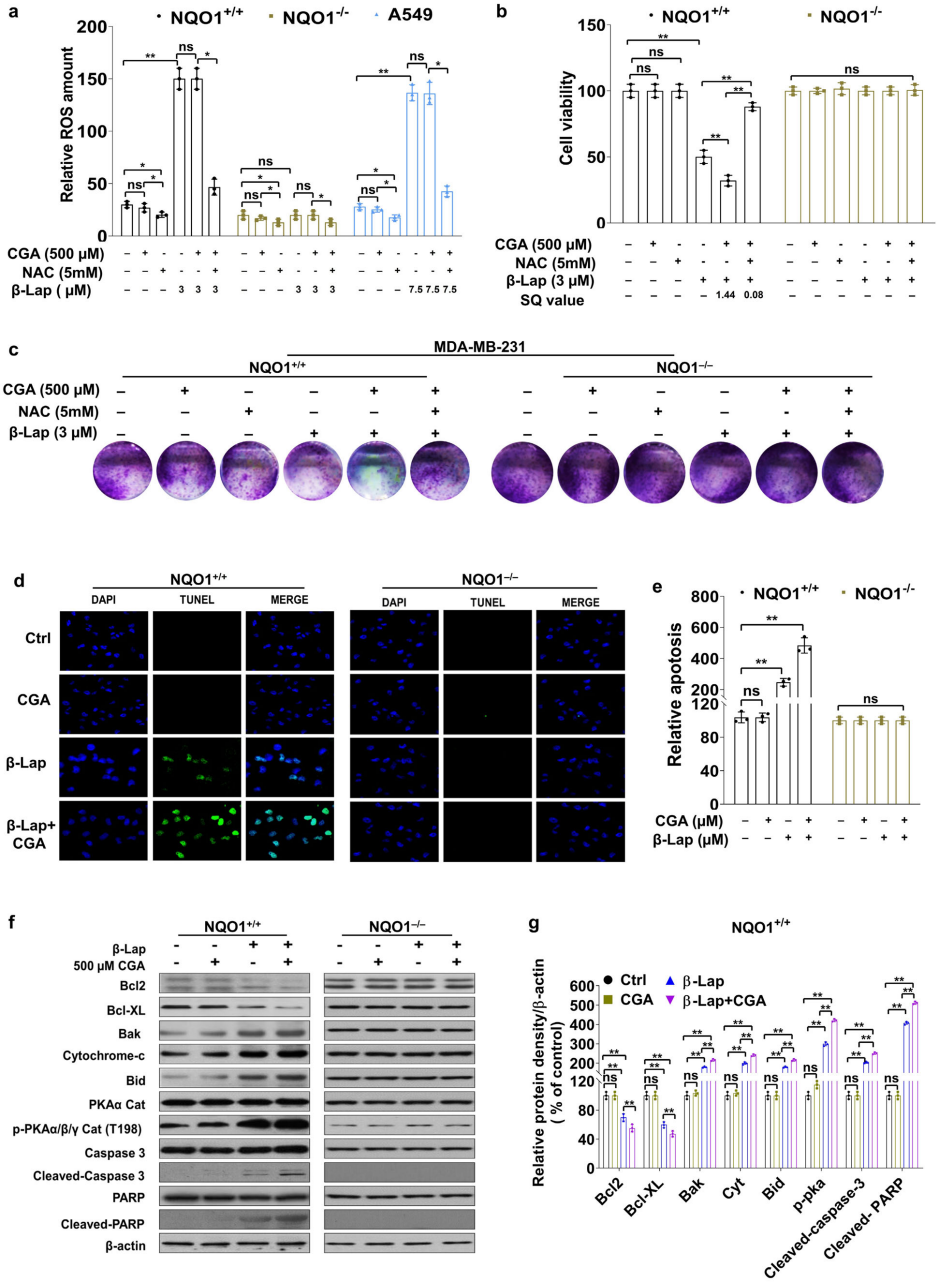
Determination of ROS dependency and PKA activation during the apoptotic cell death induced by β‐Lap and CGA in NQO1‐positive cells. (a) Cells treated with β‐Lap (0 µM or 3 µM for 231‐NQO1^+/+^ or 231‐NQO1^−/−^ cells; 0 µM or 7.5 µM for A549 cells) were incubated in the presence of 500 µM CGA or/and 5 mM *N*‐acetylcysteine (NAC) for 2 h. After further incubation for 4 h in fresh medium with 5% FBS, the relative amounts of intracellular ROS were determined by DCFDA assay. Data represent the mean (±SD) of three independent experiments (**p < 0.01). (b, c) 231‐NQO1^+/+^ or 231‐NQO1^−/−^ cells were treated with β‐Lap under the above conditions in the presence of 500 µM CGA or/and 5 mM NAC for 2 h. Cell viability and proliferation were evaluated by CCK8 assay (b) or colony formation assay (c). SQ indicates synergism quotient values. Data represent the mean (±SD) of three independent experiments (**p < 0.01). *ns* indicates not significant. (d, e) 231‐NQO1^+/+^ or 231‐NQO1^−/−^ cells were treated with 3 µM β‐Lap in the presence of 500 µM CGA for 2 h and further incubated in fresh medium with 5% FBS for 4 h. Apoptotic cells were stained for TUNEL assays using the Promega DeadEnd™ Fluorometric TUNEL system kit. Images were captured under an Olympus BX51‐DSU fluorescence microscope (d), and the relative fluorescent intensities were quantified using ImageJ software (e). Nuclei were counterstained with DAPI. Data represent the mean ± SD of three independent experiments. **p < 0.01. (f) 231‐NQO1^+/+^ or 231‐NQO1^−/−^ cells were treated with 3 µM β‐Lap in the presence of 500 µM CGA for 2 h and further incubated in fresh medium with 5% FBS for 4 h. After cell lysis, total cell extracts (30 µg) were separated by 8% or 10% SDS‐PAGE and analyzed by Western blot using primary antibodies against Bcl‐2, Bcl‐X_L_, Bak, cytochrome C, Bid, p‐PKAα/β/γ cat, PKA cat, caspase‐3, cleaved caspase‐3, PARP, and cleaved PARP. β‐actin was used as a loading control (f). (g) The relative amounts of these proteins were quantified by NIH ImageJ software and represented as graphs (g; Supporting Information: Figure 1g). CGA, chlorogenic acid; DAPI, 4',6‐diamidino‐2‐phenylindole; FBS, fetal bovine serum; PARP, poly (ADP‐ribose) polymerase; PKA, protein kinase A; ROS, reactive oxygen species; SD, standard deviation; SDS‐PAGE, sodium dodecyl sulfate‐polyacrylamide gel electrophoresis; TUNEL, terminal deoxynucleotidyl transferase dUTP nick end labeling.

CGA alone had no cytotoxic effect on the cancer cells over a range of concentrations (100–500 µM), with only a minor reduction in viable cells observed at 600 µM CGA (Figure 1e). We investigated the influence of CGA on β‐Lap‐induced cell death in these cancer cells and found that CGA treatment significantly exacerbated β‐Lap‐induced cell death in 231‐NQO1^+/+^ cells and A549 cells but not in 231‐NQO1^−/−^ cells (Figure 1f–i). The combination of β‐Lap and CGA demonstrated synergistic effects, as indicated by SQ values exceeding 1.0 for cell growth inhibition in both 231‐NQO1^+/+^ and A549 cells (Figure 1g,i). In the context of β‐Lap–dependent ROS accumulation, we examined the potential impact of CGA on ROS production in the β‐Lap–treated cancer cells. Treatment with 500 µM CGA did not affect overall intracellular ROS levels in NQO1^+/+^ and A459 cells incubated with 3 µM and 7.5 µM β‐Lap, respectively; however, ROS levels in β‐Lap–treated cells were lowered in the presence of N‐acetyl cysteine (NAC), a ROS scavenger (Figure 2a). Nevertheless, the viability of β‐Lap–treated 231‐NQO1^+/+^ cells and A549 cells was further deteriorated by CGA treatment and was subsequently recovered by NAC, as indicated in CCK8 assays and colony formation assays, whereas 231‐NQO1^−/−^ cells exhibited no change in viability under the same conditions (Figure 2b,c; Supporting Information: Figure 1a,b). Cell growth inhibition of 231‐NQO1^+/+^ and A549 cells was synergistic when CGA was combined with β‐Lap, with SQ values of 1.44 and 1.51, respectively. However, the combination of β‐Lap and CGA in the presence of NAC not only failed to enhance cell growth inhibition but also showed no synergism, with SQ values of 0.08 for 231‐NQO1^+/+^ cells and 0.04 for A549 cells (Figure 2a; Supporting Information: Figure 1a).

### 3.2 The Increase in β‐Lap‐Induced Cell Death Caused by CGA Is Associated With PKA Activation in Cancer Cells Overexpressing NQO1

In a recent study, we showed that β‐Lap can instigate apoptosis in NQO1‐overexpressing breast cancer cells through PKA activation. To delve into the intricacies of β‐Lap–mediated cell death and its relationship with CGA, we first determined relative apoptotic cell death using TUNEL assays. The numbers of TUNEL‐positive apoptotic cells were significantly augmented in populations of 231‐NQO1^+/+^ cells and A549 cells when β‐Lap was co‐administered with CGA, but this effect was not evident in 231‐NQO1^−/−^ cells (Figure 2d,e; Supporting Information: Figure 1d,e), indicating that CGA exerts a potent cytotoxic effect on NQO1‐overexpressing cells, independently of its antioxidant function.

We further compared the expression levels of apoptotic proteins following treatment with β‐Lap, CGA, or their combination in 231‐NQO1^+/+^, 231‐NQO1^−/−^, and A549 cells. As expected, compared with β‐Lap treatment alone, co‐treatment with CGA and β‐Lap in 231‐NQO1^+/+^ cells and A549 cells led to significant reductions in the levels of antiapoptotic proteins such as Bcl‐2 and Bcl‐X_L_. However, this effect was not observed in NQO1‐deficient 231‐NQO1^−/−^ cells (Figure 2f,g; Supporting Information: Figure 1e–g). By contrast, compared with β‐Lap alone, co‐treatment with CGA and β‐Lap in 231‐NQO1^+/+^ cells and A549 cells substantially increased the expression of proapoptotic proteins, including Bak, Bid, and cytochrome *c*, as well as caspase‐3 activation (cleaved caspase‐3) and PARP cleavage, but this effect was not observed in 231‐NQO1^−/−^ cells (Figure 2f,g; Supporting Information: Figure 1e–g). Interestingly, compared with β‐Lap alone, co‐treatment with both CGA and β‐Lap resulted in a significant increase of PKA activation (phosphorylated‐PKAα/β/γ at T198) in 231‐NQO1^+/+^ cells and A549 cells but not in 231‐NQO1^−/−^ cells (Figure 2f,g; Supporting Information: Figure 1e–g). These results suggest that PKA activation plays a role in CGA‐dependent exacerbation of β‐Lap‐induced apoptosis in 231‐NQO1^+/+^ cancer cells.

### 3.3 CGA Increases PKA Activity During β‐Lap‐Induced Cell Death

**Figure 3:**
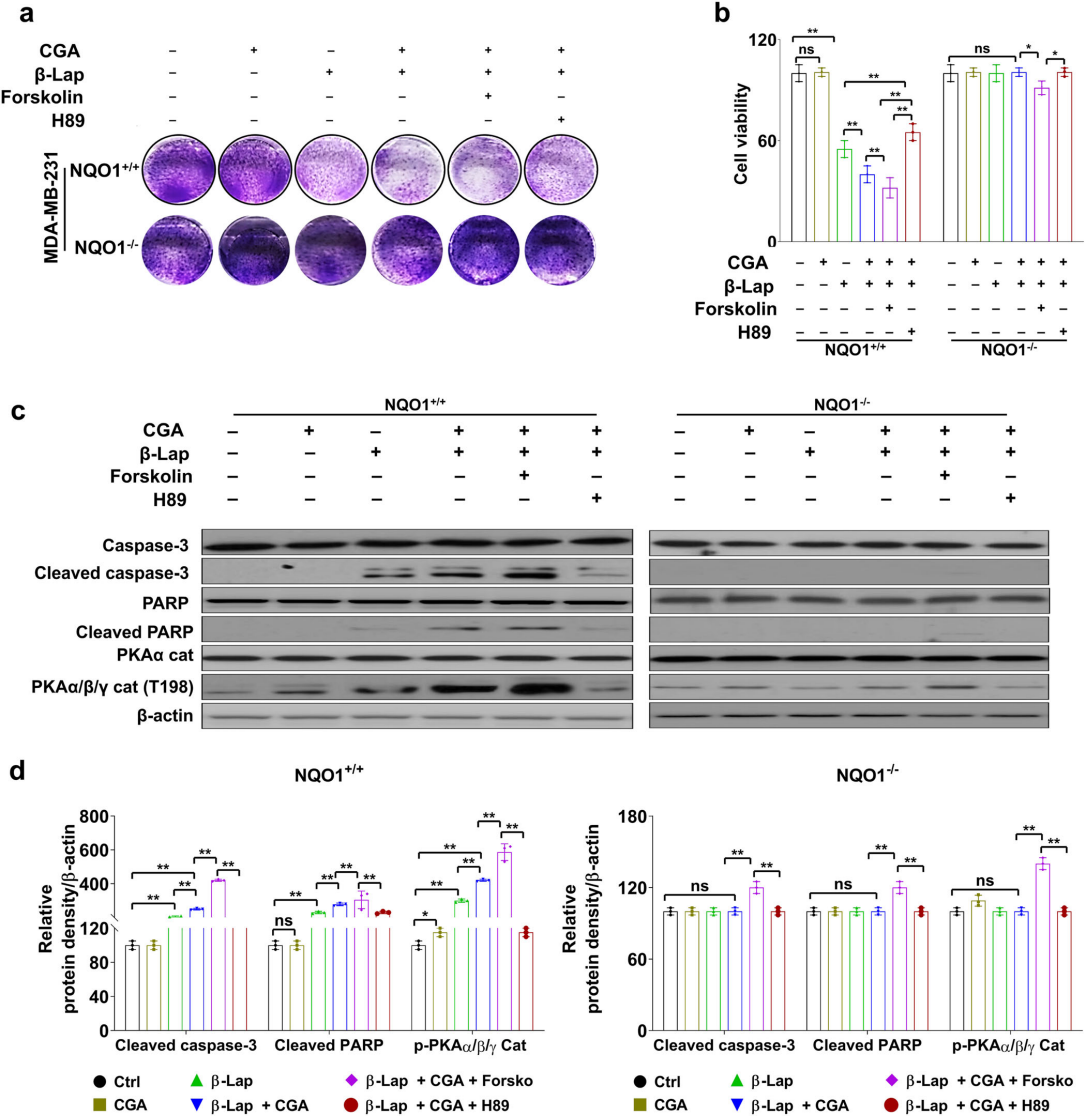
PKA‐dependent regulation of apoptotic cell death by β‐Lap and CGA. (a, b) 231‐NQO1^+/+^ or 231‐NQO1^−/−^ cells were treated with 3 µM β‐Lap alone or in combination with 500 µM CGA in the presence of PKA activator (10 µM forskolin) or PKA inhibitor (5 µM H89) for 2 h. The cells were then further incubated in fresh medium with 5% FBS containing either PKA activator or PKA inhibitor for 4 h. Cell proliferation was assessed by clonogenic assay (a), and cell viability was determined by CCK‐8 assay (b). Data represent the mean (±SD) of three independent experiments (*p < 0.05 and **p < 0.01). Cell images were captured under a bright‐field microscope (100× magnification). (c, d) Western blot. Total cell extracts (30 µg) of treated 231‐NQO1^+/+^ and 231‐NQO1^−/−^ cells were separated by 8% or 10% SDS‐PAGE and analyzed by western blot using primary antibodies against p‐PKAα/β/γ cat, PKA cat, caspase‐3, cleaved caspase‐3, PARP, and cleaved PARP (c). β‐actin was used as a loading control. The relative amounts of these proteins were quantified using ImageJ software and represented as a graph (d). Data represent the mean (±SD) of three independent experiments (*p < 0.05 and **p < 0.01). CCK‐8, Cell Counting Kit‐8; CGA, chlorogenic acid; FBS, fetal bovine serum; PARP, poly (ADP‐ribose) polymerase; PKA, protein kinase A; SD, standard deviation; SDS‐PAGE, sodium dodecyl sulfate‐polyacrylamide gel electrophoresis.

Our results suggest that the PKA signaling pathway is closely associated with β‐Lap‐induced cell death in 231‐NQO1^+/+^ cells and A549 cells. Previous studies have suggested that PKA signaling may serve as a potential activator of apoptosis (de Joussineau et al. 2014, Keshwani et al. 2015). To investigate the possible role of PKA activation in cell death induced by β‐Lap and CGA, we further treated the cancer cell lines with PKA activator (forskolin) or inhibitor (H89). Notably, both 231‐NQO1^+/+^ cells and A549 cells exhibited a significant reduction in viability when treated with the combination of β‐Lap, CGA, and forskolin. Their survival rates were restored by the introduction of H89, as indicated by CCK8 and clonogenic assays. However, this PKA‐dependent cell viability was not observed in 231‐NQO1^−/−^ cells (Figure 3a,b; Supporting Information: Figure 2a,b). These findings strongly suggest that PKA activity is essential for enhanced cell death induced by CGA in β‐Lap–treated cancer cells.

Next, we delved further into cell death‐related protein expression and PKA activation in 231‐NQO1^+/+^ cells, 231‐NQO1^−/−^ cells, and A549 cells subjected to co‐treatment with β‐Lap and CGA in the presence of PKA inhibitor or activator. As expected, proapoptotic signals such as caspase‐3 activation and PARP cleavage, as well as PKA activation (p‐PKAα/β/γ at T198), exhibited gradual increases in 231‐NQO1^+/+^ cells and A549 cells when the cells were treated with a combination of β‐Lap and CGA in the presence of forskolin; however, the expression of these apoptotic signals and PKA activation were reversed in the presence of H89 (Figure 3c,d; Supporting Information: Figure 2c,d). By contrast, this PKA‐dependent regulation of apoptotic proteins was not observed in 231‐NQO1^−/−^ cells, although forskolin had a slight effect in increasing PKA activity (Figure 3c,d and Supporting Information: Figure 2c,d).

### 3.4 PKA Activation Induced by CGA and β‐Lap Inhibits Autophagy Via LC3 Phosphorylation at the Serine 12 Residue

**Figure 4:**
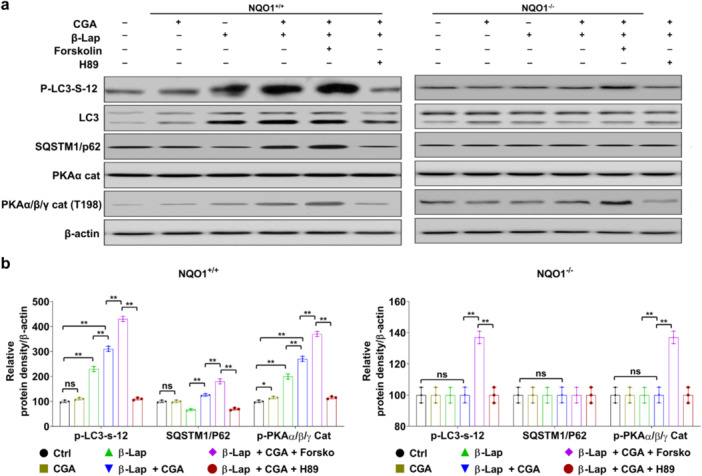
PKA activation induced by the combination of β‐Lap and CGA was associated with autophagy inhibition through phosphorylation of LC3 at the serine 12 residue. (a, b) 231‐NQO1^+/+^ or 231‐NQO1^−/−^ cells were treated with 3 µM β‐Lap alone or in combination with 500 µM CGA in the presence of PKA activator (10 µM forskolin) or PKA inhibitor (5 µM H89) for 2 h. The cells were then further incubated in fresh medium with 5% FBS containing either PKA activator or PKA inhibitor for 4 h. The protein expression level was assessed by western blot. Total cell extracts (30 µg) of treated 231‐NQO1^+/+^ or 231‐NQO1^−/−^ cells were separated by 8% or 10% SDS‐PAGE. Expression of phosphorylated‐LC3 (p‐LC3‐Ser‐12), LC3, SQSTM1/P62, p‐PKAα/β/γ cat, and PKA was analyzed by western blot (a). β‐actin was used as a loading control. The relative levels of these proteins were quantified and represented as a graph (b). Data represent the mean (±SD) of three independent experiments (*p < 0.05 and **p < 0.01). CGA, chlorogenic acid; FBS, fetal bovine serum; PKA, protein kinase A; SD, standard deviation; SDS‐PAGE, sodium dodecyl sulfate‐polyacrylamide gel electrophoresis.

The combination treatment of β‐Lap and CGA substantially increased PKA activity in 231‐NQO1^+/+^ cells and A549 cells. Furthermore, autophagy activities, as indicated by p62 levels, were reduced by β‐Lap treatment alone in 231‐NQO1^+/+^ cells and A549 cells; however, when β‐Lap was combined with CGA, p62 levels were slightly induced, likely because of stimulation of PKA activity (Figure 4a,b; Supporting Information: Figure 3a,b). Notably, when these cells were further treated with PKA activator (forskolin) or inhibitor (H89) in conjunction with β‐Lap and CGA, the autophagy levels exhibited significant changes. In the presence of forskolin, the autophagy activities were significantly decreased in cancer cells treated with β‐Lap and CGA. Conversely, the addition of H89 alongside β‐Lap and CGA in 231‐NQO1^+/+^ cells and A549 cells resulted in upregulation of autophagy activities, as indicated by either the protein level of LC3‐II/p62, but this effect was not observed in 231‐NQO1^−/−^ cells (Figure 4a,b; Supporting Information: Figure 3a,b).

Remarkably, when β‐Lap and CGA were combined with further forskolin treatment in 231‐NQO1^+/+^ cells and A549 cells, we observed a substantial increase in LC3 phosphorylation at the serine 12 residue, leading to the inhibition of autophagy (Figure 4a,d; Supporting Information: Figure 3a,b). These findings suggest that the heightened PKA activity induced by β‐Lap and CGA in NQO1‐overexpressing cancer cells could modulate autophagy through LC3 phosphorylation at the serine 12 residue.

### 3.5 Autophagy Modulation by CGA During β‐Lap‐Induced Cell Death Is PKA‐Dependent but MTOR‐Independent

**Figure 5:**
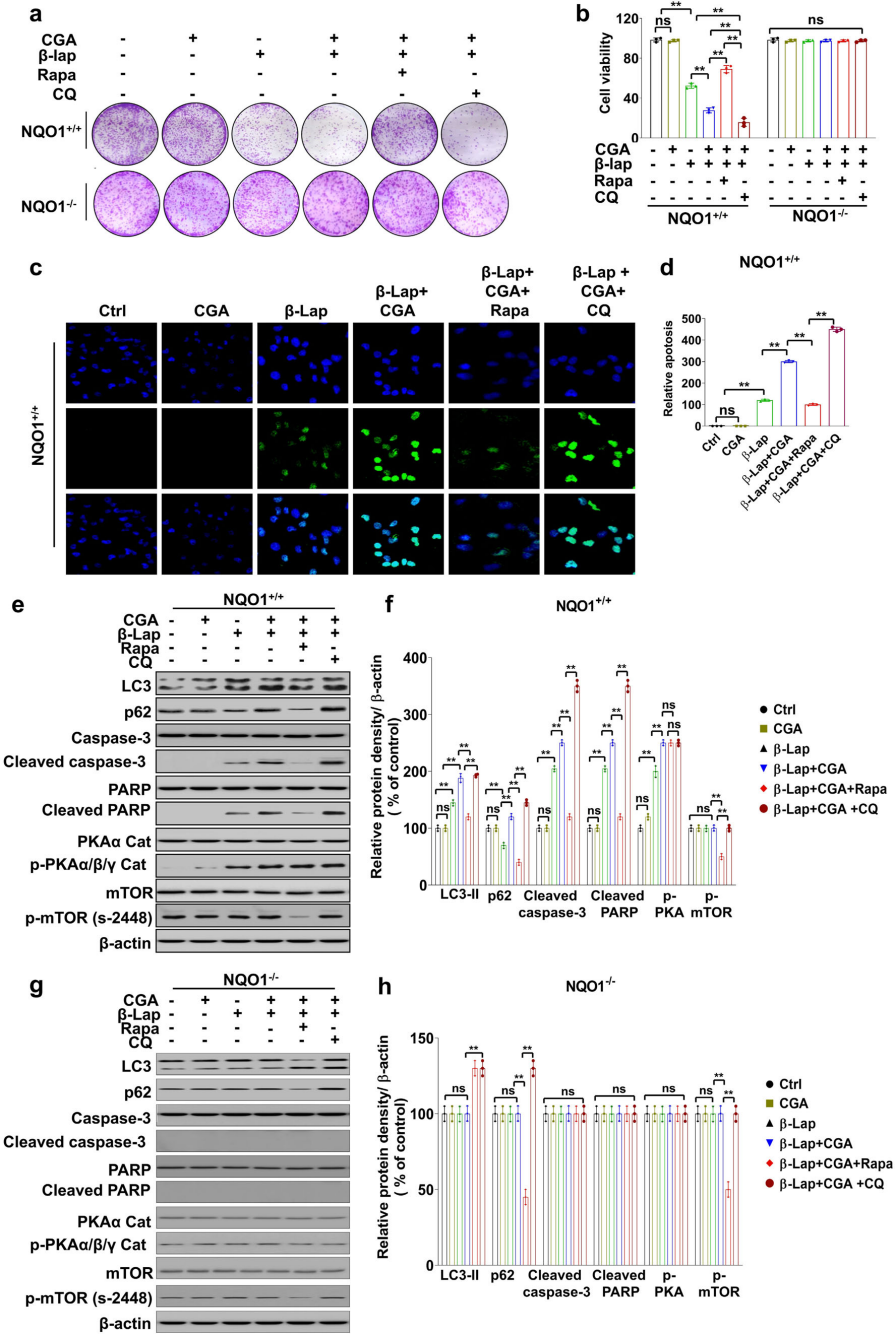
Autophagy activation reduced β‐Lap‐induced cell death in combination with CGA, independently of mTORC. (a, b) 231‐NQO1^+/+^ or 231‐NQO1^−/−^ cells were treated with 3 µM β‐Lap alone or in combination with 500 µM CGA in the presence of autophagy activator (100 nM Rapa) or autophagy inhibitor (15 µM CQ) for 2 h. Then, the cells were incubated in fresh medium with 5% FBS containing the autophagy modulator for 4 h. Cell proliferation and viability were determined by clonogenic analysis (a) and CCK‐8 assay (b), respectively. Data represent the mean (±SD) of three independent experiments (*p < 0.05 and **p < 0.01). After cell staining with crystal violet, images of colony formation were captured by a digital camera. (c, d) TUNEL analysis. 231‐NQO1^+/+^ cells were treated as described above. Apoptotic cells were stained using the Promega DeadEnd™ Fluorometric TUNEL system kit. Images were captured under a fluorescent microscope (Olympus BX51‐DSU) (c), and the relative levels of TUNEL‐positive nuclei counterstained with DAPI were represented as a graph (d). Data represent the mean ± SD of three independent experiments. **p < 0.01. (e) Western blot. 231‐NQO1^+/+^ and 231‐NQO1^−/−^ cells were treated with β‐Lap (3 µM) or/and CGA (500 µM) in the presence of 100 nM rapamycin or 15 µM CQ for 2 h. Then, the cells were further incubated in fresh medium with 5% FBS containing autophagy activator or inhibitor for 4 h. Total cell extracts (30 µg) were separated by 8% or 12% SDS‐PAGE and analyzed by Western blot using primary antibodies against p‐LC3(s‐12), LC3, SQSTM1/P62, caspase‐3, cleaved caspase‐3, PARP, cleaved PAR, p‐PKAα/β/γ cat, PKA, p‐mTOR (s‐2448), and mTOR. β‐actin was used as a loading control. The relative levels of the indicated proteins in 231‐NQO1^+/+^ and 231‐NQO1^−/−^ cells were quantified and represented as graphs (f, h). CGA, chlorogenic acid; DAPI, 4',6‐diamidino‐2‐phenylindole; FBS, fetal bovine serum; PARP, poly (ADP‐ribose) polymerase; SD, standard deviation; SDS‐PAGE, sodium dodecyl sulfate‐polyacrylamide gel electrophoresis; TUNEL, terminal deoxynucleotidyl transferase dUTP nick end labeling.

We conducted CCK8 and colony formation assays to further investigate cell viability and its correlation with autophagy in 231‐NQO1^+/+^ cells, 231‐NQO1^−/−^ cells, and A549 cells co‐treated with β‐Lap and CGA in the presence of rapamycin (an autophagy activator) or CQ (an autophagy inhibitor). As expected, combined treatment with β‐Lap and CGA substantially reduced the viability of 231‐NQO1^+/+^ cells and A549 cells, whereas 231‐NQO1^−/−^ cells showed no such effect (Figure 5a,b, Supporting Information: Figure 4a,b). Interestingly, further induction of autophagy with rapamycin significantly restored cell viability that had been reduced by the combination of β‐Lap and CGA. By contrast, inhibition of autophagy with CQ in these cells exacerbated the decline in viability (Figure 5a,b, Supporting Information: Figure 4a,b). Moreover, TUNEL assays of these cancer cells revealed a corresponding pattern of apoptotic cell death dependent on co‐treatment with β‐Lap, CGA, and either an autophagy activator or an autophagy inhibitor (Figure 5c,d, Supporting Information: Figure 4c,d). These results indicate that autophagy can serve as a crucial modulator of cell viability in NQO1‐expressing cancer cells during chemotherapy.

Levels of apoptosis‐related proteins were significantly decreased upon activation of autophagy with rapamycin in NQO1‐expressing cancer cells co‐treated with β‐Lap and CGA. By contrast, the levels of these apoptotic proteins were markedly increased in these cells upon inhibition of autophagy in the presence of CGA and β‐Lap (Figure 5e–h; Supporting Information: Figure 4e,f). Surprisingly, the phosphorylated PKA level in both NQO1‐expressing cancer cell lines remained unchanged following autophagy modulation, whether activation by rapamycin or inhibition by CQ, indicating that PKA can regulate autophagy but not vice versa, meaning that autophagy activation was not associated with the direct activation of PKA. However, these effects were not observed in NQO1‐deficient cells (Figure 5e–h; Supporting Information: Figure 4e,f).

Additionally, phosphorylated mTOR (p‐mTOR) levels did not change with β‐Lap treatment alone or in combination with CGA in any of the cancer cells, despite the reduction in p‐mTOR levels induced by the mTOR inhibitor rapamycin (Figure 5e–h; Supporting Information: Figure 4e,f). These results suggest that PKA activation might be employed as a therapeutic target to modulate autophagy in an mTOR‐independent manner.

## 4 Discussion

In this study, we showed that β‐Lap induces apoptotic cell death through PKA activation in human cancer cells, and this effect is dependent on the presence of NQO1. Furthermore, combination treatment with β‐Lap and CGA significantly enhanced cancer cell death. In breast cancer MDA‐MB‐231 cells or lung cancer A549 cells that overexpress NQO1, co‐treatment with β‐Lap and CGA led to a remarkable increase in PKA activation (phosphorylation at Thr198) and subsequently inhibited autophagy by inducing LC3 phosphorylation at serine 12.

Previous studies have established that β‐Lap‐induced cell death primarily relies on the generation of ROS through an intracellular metabolic pathway involving a futile cycle, specifically in an NQO1‐dependent manner (Lendi et al. 1995). We also observed a gradual dose‐dependent increase in intracellular ROS levels when 231‐NQO1^+/+^ cells were exposed to β‐Lap. In particular, PKA activation during β‐Lap‐induced death of 231‐NQO1^+/+^ cells was significantly augmented when CGA was added. These findings suggest that PKA activation induced by the combination of β‐Lap and CGA might play a pivotal role in controlling cell proliferation via autophagy. PKA activators such as forskolin further intensified cell death induced by β‐Lap and CGA, whereas PKA inhibitors, including H89, substantially improved cell survival, even in 231‐NQO1^+/+^ cells and A549 cells treated with β‐Lap and CGA. Furthermore, this autophagy inhibition does not rely on mTOR activity. Taken together, these findings suggest that activation of PKA activity by β‐Lap and CGA is crucial for the synergetic induction of apoptotic cell death via autophagy inhibition in breast and lung cancer cells that overexpress NQO1. Based on these findings, we propose that combination therapy targeting autophagy holds great promise for the development of new therapeutic approaches for cancers that exhibit resistance to drug treatment.

β‐Lap, a natural compound originated from the lapacho tree (*Tabebuia avellanedae*), is commonly employed in the treatment of various types of cancers that overexpress NQO1. Previous studies have primarily focused on enhancing the effectiveness of β‐Lap by elevating intracellular ROS levels in NQO1‐ocerexpressing cancer cells (Cao et al. 2014, Lee et al. 2014). In contrast to that approach, our study reveals a novel mechanism for enhancing the therapeutic potential of β‐Lap. We demonstrated that the potential anticancer effect of β‐Lap was augmented via autophagy inhibition by CGA, which is crucial for combating drug resistance. Notably, our findings suggest that the enhancement of β‐Lap‐induced cell death by CGA is likely independent of NQO1 expression and ROS levels in cancer cells (Figure 2a). These discoveries contribute to our understanding of how cancer cells can be selectively targeted with the combination of CGA and β‐Lap, suggesting new strategies to improve the anticancer efficacy of potential drugs while minimizing adverse effects on normal cells.

PKA plays a multifaceted role in tumorigenesis, either promoting or inhibiting apoptosis and regulating autophagy, among other mechanisms that influence cell survival or death. Previous studies have shown that PKA can inhibit autophagy by directly phosphorylating key autophagy proteins such as ULK1 and LC3 (Holloway and Marignani 2021). In our investigation, we demonstrated that increased PKA activity inhibited autophagy in cancer cells subjected to CGA and β‐Lap co‐treatment (Figure 4). Notably, whereas β‐Lap treatment alone significantly induced autophagy, the combination of β‐Lap with CGA led to inhibition of the autophagy process. This outcome may be attributed to the PKA‐dependent phosphorylation of LC3 in cancer cells treated with CGA and β‐Lap. Based on these findings, we propose that targeting PKA activity and modulating autophagy can serve as a valuable therapeutic approach to overcome drug resistance in cancer and enhance the effectiveness of chemotherapy.

Autophagy, which is often triggered by anticancer drugs, plays a pivotal role in regulating the resistance of cancer cells to chemotherapy (Liu et al. 2020). Therefore, adopting combination strategies to modulate autophagy might offer therapeutic advantages in terms of overcoming drug resistance and enhancing the efficacy of antitumor treatments. Recent studies have proposed autophagy as a potential mechanism of drug resistance in a wide spectrum of cancer cells, including tamoxifen‐resistant breast cancer cells (Ho and Gorski 2019, Lee et al. 2018, Samaddar et al. 2008). As a result, several endeavors have focused on developing autophagy inhibitors in combination with other anticancer drugs to combat drug resistance in chemotherapy. For instance, autophagy inhibitors such as CQ have shown promise in augmenting the cytotoxic effect of temozolomide in metastatic melanoma by diminishing autophagy‐dependent resistance (Ryabaya et al. 2017). Conversely, certain anticancer therapeutic agents exhibit cytoprotective effects by activating autophagy in lung cancer cells (Gorzalczany et al. 2011, Han et al. 2011). Notably, breast cancer cells treated with tamoxifen display increased metastatic traits due to autophagy‐induced drug resistance; however, inhibiting autophagy can reverse this resistance (Cook et al. 2011, Das et al. 2019). The development of combination interventions that modulate autophagy is becoming a novel therapeutic strategy in cancer treatment (Cerniglia et al. 2012, Liu et al. 2015, Rahim and Strobl 2009). Furthermore, autophagy inhibition has been found to enhance apoptotic pathways in chemoresistant tumors PMID: 25712477, PMID: 25842161. Conversely, mTORC1 inhibitors such as rapamycin or resveratrol, which activate autophagy, are also employed in the treatment of breast cancer. This suggests that induced autophagy may be implicated in the regulation of cell death (Alayev et al. 2015, Bahar et al. 2024). Prolonged autophagy is additionally linked to a type of cell death known as type II cell death, which contributes to cancer treatment by interacting with apoptosis (Bahar et al. 2022, Kroemer and Levine 2008, Zada et al. 2021).

The development of breast cancer therapy involves targeting the mTOR pathway, not only as a standalone treatment but also in combination with other treatments. While more than 80 clinical trials have explored the use of mTOR inhibitors in cancer patients, it is evident that many cancer cells develop resistance to these inhibitors (Del Campo et al. 2016, Powles et al. 2016). Therefore, alternative strategies focusing on mTOR‐independent autophagy activation might offer significant benefits for cancers displaying drug resistance. In this context, our study demonstrates that combined treatment with CGA and β‐Lap inhibits autophagy by a PKA‐dependent mechanism that operates independently of mTORC activity. Remarkably, autophagy activation with rapamycin, an mTORC inhibitor, reduced cell death associated with CGA and β‐Lap co‐treatment, whereas autophagy inhibition with CQ significantly enhanced this cell death. These findings underscore the essential role of autophagy in the effectiveness of CGA and β‐Lap combination therapy.

**Figure 6:**
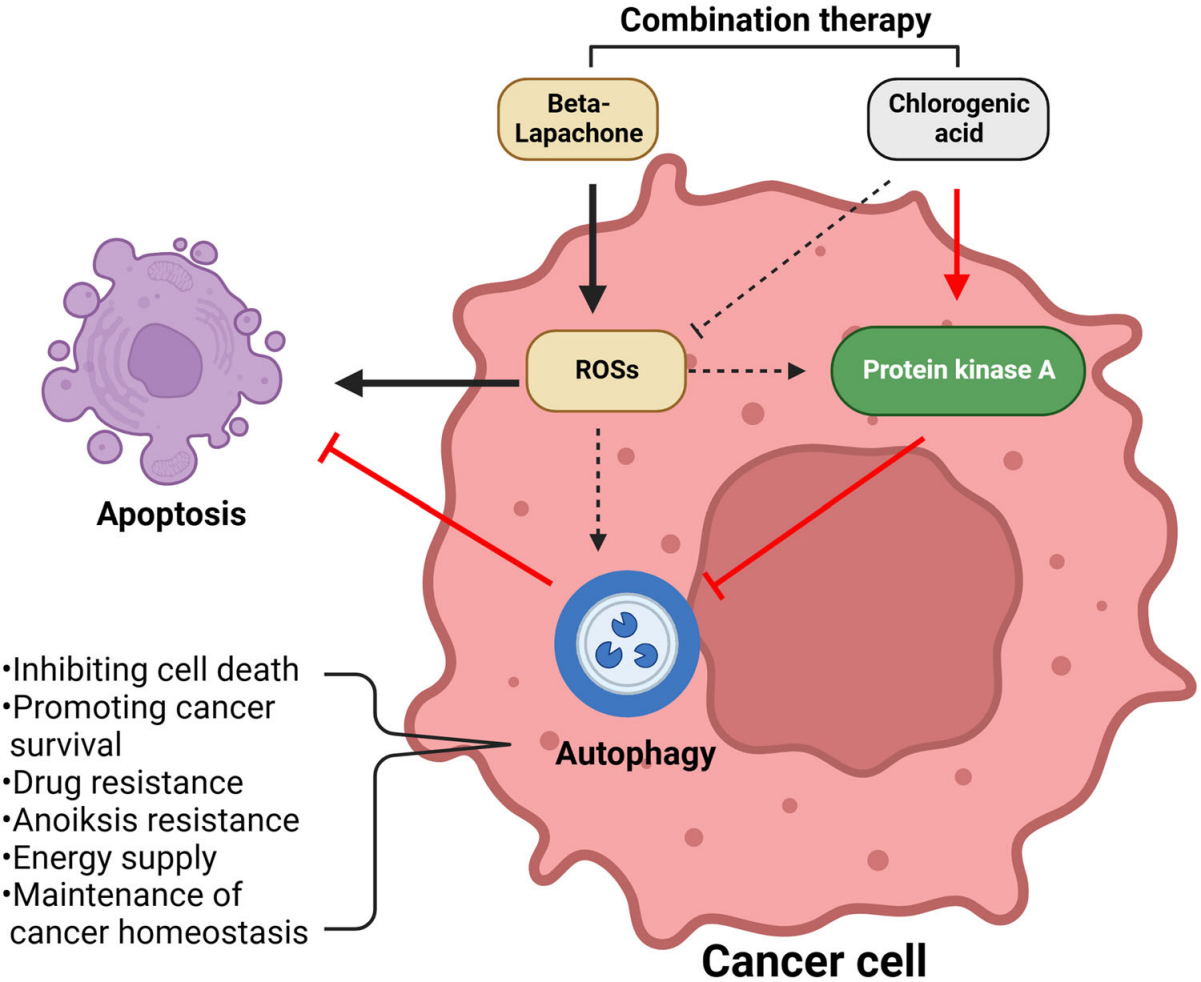
A graphical representation of the modulation of apoptosis induced by β‐Lap in combination with CGA in cancer cells: a novel combination cancer therapy. Activation of the PKA signaling pathway induced by CGA is associated with elevated β‐Lap–dependent apoptotic cell death due to ROS production in cancer cells that highly express NQO1. PKA activation inhibits autophagy, leading to cell death. Solid lines (*black*, followed by β‐Lap; *red*, followed by chlorogenic acid) present the possible mechanisms suggested in this study. Dotted lines indicate potential roles in cancer cells. This figure was created with www.Biorender.com. CGA, chlorogenic acid; PKA, protein kinase A.

Autophagy induced by starvation produces similar effects in CGA/β‐Lap–treated cancer cells. Beyond its involvement in cell death, autophagy is known to support the survival of cancer cells under conditions of nutrient deprivation or therapeutic stress. Therefore, the role of autophagy in cancer is remarkably diverse and contingent on various cellular contexts. In addition to the cross‐regulation mechanisms discussed here, there is a pressing need for in‐depth exploration of other mechanisms based on the intricate crosstalk between autophagy and apoptosis, including PKA‐dependent regulation of cell death and the interplay of the mTORC pathway with resistance to anticancer drugs (Figure 6). Comprehending these multifaceted molecular aspects of cancer resistance pathways will open the door to new opportunities for selective therapeutic interventions against various cancer types, potentially leading to the development of successful anticancer treatments.

## 5 Conclusion

This study addresses the challenge of drug resistance in cancer treatment by exploring autophagy inhibition as a potential strategy. We found that CGA significantly enhances β‐Lap‐induced cell death in cancer cells. This increased apoptosis is linked to the activation of PKA in β‐Lap‐treated cells, independent of CGA's antioxidant properties. PKA activation inhibits autophagy by phosphorylating LC3 at serine 12, regardless of mTORC activity. This mechanism is particularly effective in NQO1‐overexpressing cancer cells. Our findings suggest that the combination of β‐Lap and CGA could be a novel therapeutic approach to overcome drug resistance in cancer cells.

## Author Contributions

Sahib Zada conceptualized, conducted the experiments, analyzed the results, and wrote the manuscript. Md Entaz Bahar edited the manuscript and conducted experiments. Wanil Kim provided experimental resources, and edited the manuscript. Deok Ryong Kim provided financial support, supervised the study, and wrote the manuscript. All authors reviewed the manuscript.

## Conflicts of Interest

The authors declare no conflicts of interest.

## Supporting information

Supporting information.

## Data Availability

The data that support the findings of this study are available from the corresponding author upon reasonable request. The datasets used and/or analyzed during the current study are available from the corresponding author on reasonable request.
