# Supplementary material for: Chlorogenic Acid Enhances Beta‐Lapachone‐Induced Cell Death by Suppressing Autophagy in NQO1‐Positive Cancer Cells
Source: Cell Biol Int. 2025 Feb 27;49(5):555–69. doi: 10.1002/cbin.70006 (PMC11994878; doi:10.1002/cbin.70006)
Supplement: Supplementary file 1 — Supporting information. [file CBIN-49-555-s001.docx]

**Supplementary files**

**
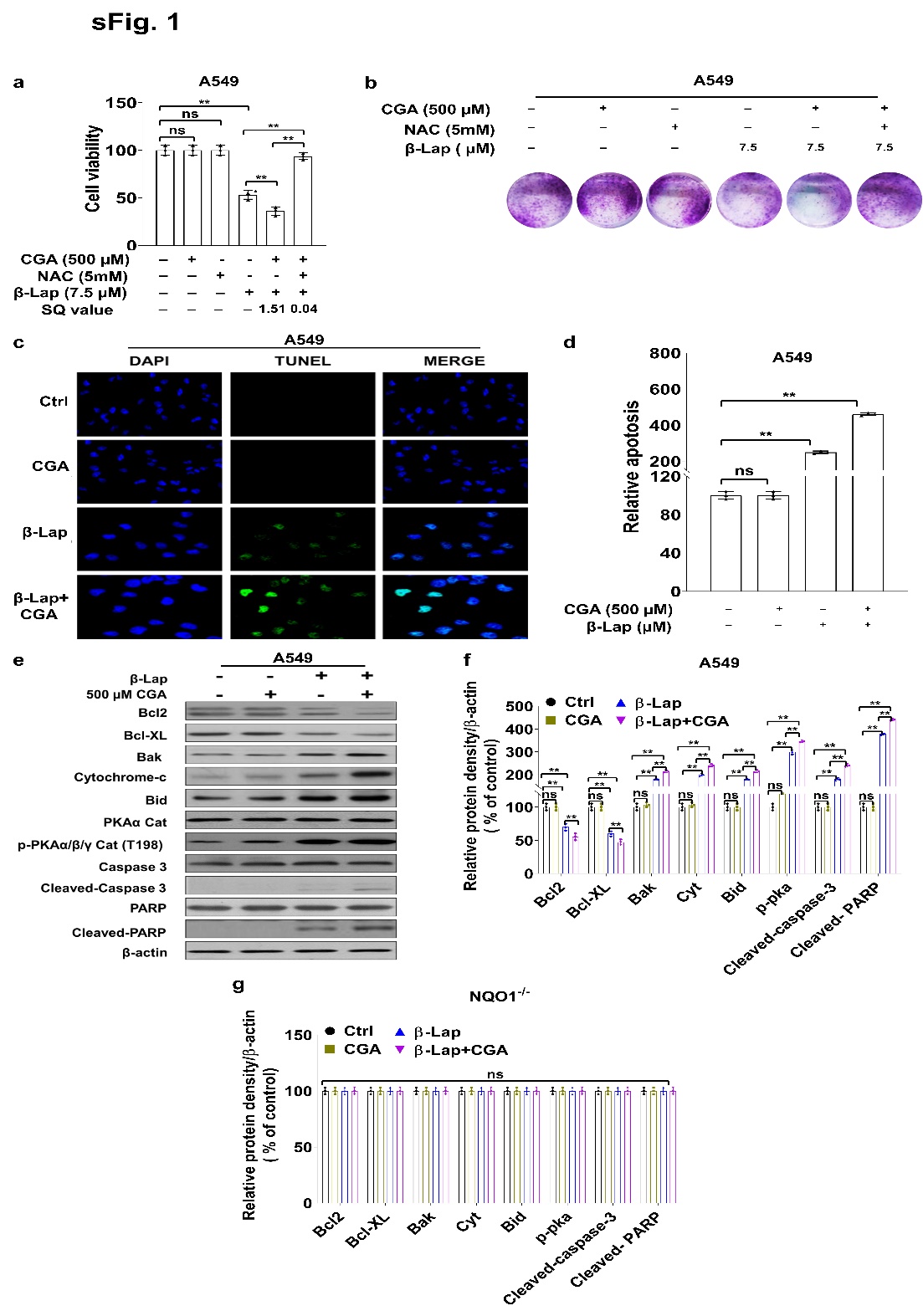
**

**sFig. 1. PKA activation during the apoptotic cell death induced by β-Lap and CGA in A549 lung cancer cells.** **(a, c)** **Cell viability and proliferation**. A549 cells with β-Lap (7.5 µM) were incubated in the presence 500 µM CGA or/and 5 mM N-acetylcysteine (NAC) for 2 h. Cell viability and proliferation were evaluated with either CCK8 assay (**a**) or colony formation (**b**) as described. SQ indicate synergism quotient values. Data represent the mean (± SD) of three independent experiments (^**^*p* < 0.01). *ns* indicates not significant. **(c, d) TUNEL assay.** A549 cells were treated with 7.5 µM β-Lap in the presence of 500 µM CGA for 2 h and further incubated in the fresh medium with 5 % FBS for 4 h. Apoptotic cells were stained by TUNEL assays using the Promega DeadEnd™ Fluorometric TUNEL system kit. The images were captured under an Olympus BX51-DSU fluorescence microscope (**c**), and the relative fluorescent intensities were quantified by a NIH ImageJ software (**d**). Nuclei were counterstained with DAPI. Data represent the mean ± SD of three independent experiments. ^**^*p* < 0.01. **(e-g) Western blots.** Total cell extracts (30 µg) of A549 cells treated previously were separated on a 8% or 10% SDS-PAGE and analyzed by Western blotting using primary antibodies against proteins (Bcl-2, Bcl-X_L_, Bak, cytochrome C, Bid, p-PKAα/β/γ cat, PKA cat, caspase-3, cleaved caspase-3, PARP, and cleaved PARP). β-actin was used as a loading control (**e**). The relative amounts of these proteins were quantified by NIH ImageJ software and (**f**). (**g**) Quantification of Western blots in 231-NQO1^-/-^ cells (**Figure 1f**).


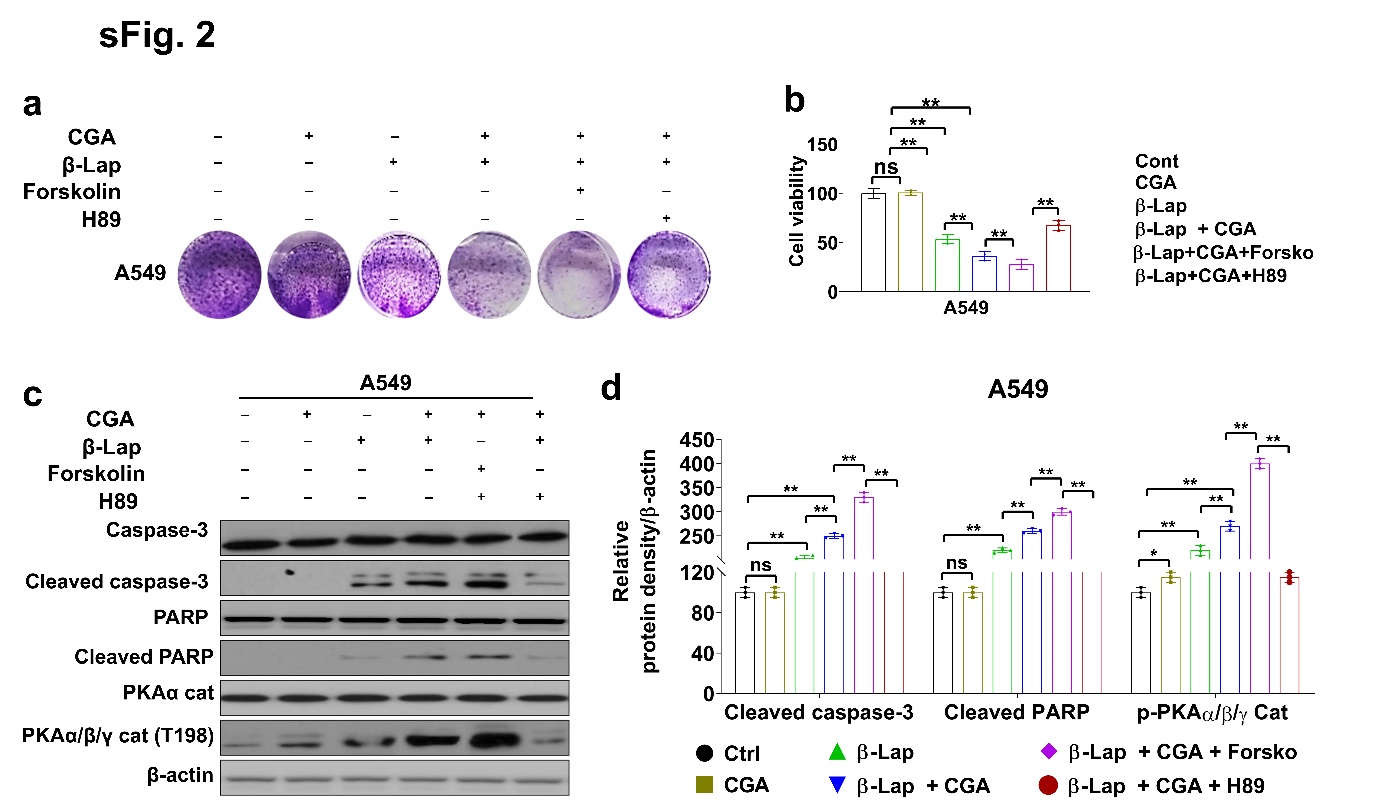


**sFig. 2. Apoptotic cell death by β-Lap and CGA in A549 cells in a PKA-dependent manner. (a, b)** **Cell viability and proliferation**. A549 cells were treated with 7.5 µM β-Lap alone or in combination with 500 µM CGA in the presence of PKA activator (10 µM Forskolin) or PKA inhibitor (5 µM H89) for 2 h, and further incubated in the fresh medium with 5% FBS containing either PKA activator or inhibitor for 4 h. Cell proliferation was assessed by the clonogenic assay (**a**) and cell viability was determined by CCK-8 assays (**b**). Data represent the mean (± SD) of three independent experiments (^*^*p* < 0.05 and ^**^*p* < 0.01). Cell images were captured under a bright-field microscope (100× magnification). (**c, d**) **Western blot**. Total cell extracts (30 µg) of A549 cells treated as mentioned above were separated on an 8% or 10% SDS-PAGE and analyzed by Western blotting using primary antibodies against p-PKAα/β/γ cat, PKA cat, caspase-3, cleaved caspase-3, PARP, and cleaved PARP (**c**). β-actin was used as a loading control. The relative amount of these proteins was quantified using a NIH ImageJ software and represented as a graph (**d**). Data represent the mean (± SD) of three independent experiments (^*^*p* < 0.05 and ^**^*p* < 0.01). *ns* indicates not significant.


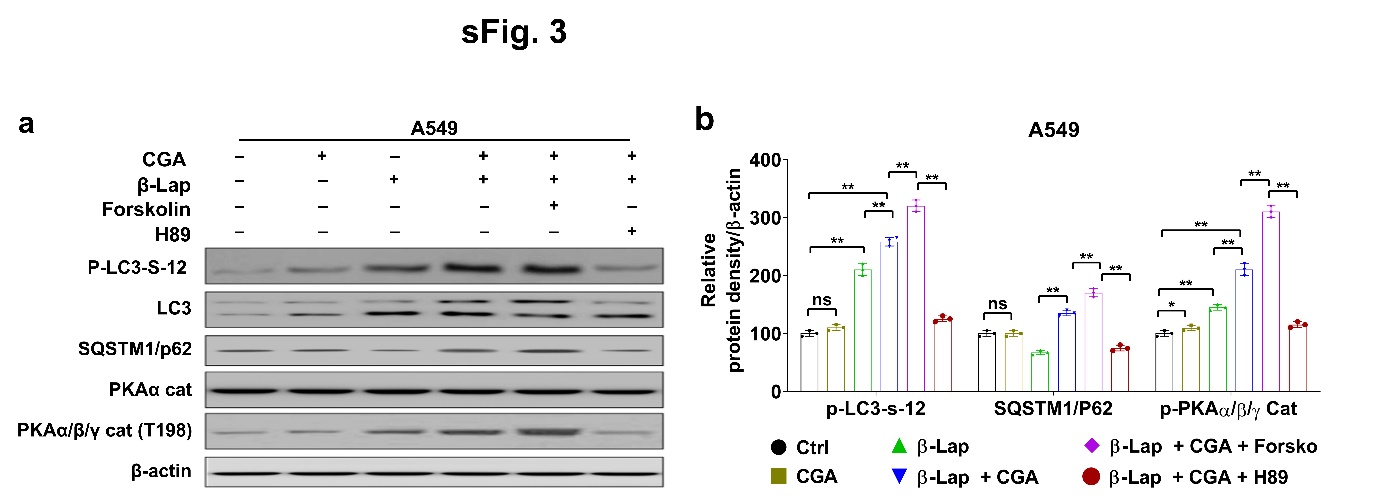


**sFig.3. PKA activation by the β-Lap and CGA combination in A549 cells was associated with autophagy inhibition.** Total cell extracts (30 µg) of A549 cells treated as described above were separated on an 8% or 10% SDS-PAGE. Expression of phosphorylated-LC3 (p-LC3-Ser-12), LC3, SQSTM1/P62, p-PKAα/β/γ cat and PKA was analyzed by Western blotting (**a**). β-actin was used as a loading control. The relative levels of these proteins were quantified represented as a graph (**b**). Data represent the mean (± SD) of three independent experiments (^*^*p* < 0.05 and ^**^*p* < 0.01). *ns* indicates not significant.


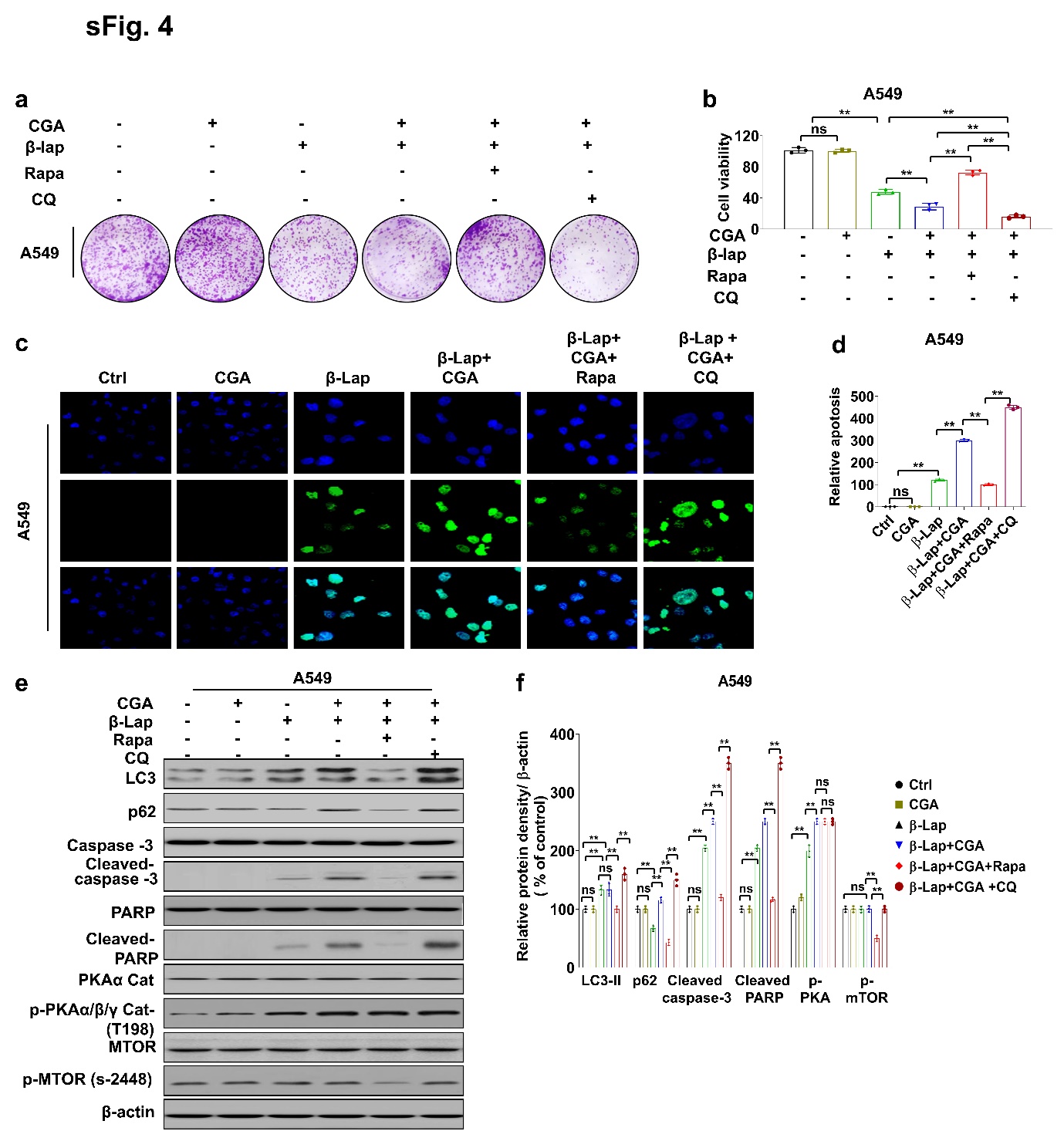


**sFig. 4. Autophagy modulated β-Lap-induced cell death in combination with CGA in A549 cells.** **(a, b)** **Cell viability and proliferation**. A549 cells were treated with 7.5 µM β-Lap alone or in combination with 500 µM CGA in the presence of autophagy activator (100 nM Rapa) or autophagy inhibitor (15 µM CQ) for 2 h, and additionally incubated in the fresh medium with 5% FBS containing this autophagy modulator for 4 h. Cell proliferation and viability were determined by the chlonogenic analysis (**a**) and CCK-8 assay (**b**), respectively. Data represent the mean (± SD) of three independent experiments (^*^*p* < 0.05 and ^**^*p* < 0.01). *ns* indicates not significant. **(c, d) TUNEL analysis.** A549 cells were treated as described above. Apoptotic cells were stained using the Promega DeadEnd™ Fluorometric TUNEL system kit. The images were captured under a fluorescent microscope (Olympus BX51-DSU) (**c**) and, the relative levels of TUNEL-positive nuclei counterstained with DAPI were represented as a graph (**d**). Data represent the mean ± SD of three independent experiments. ^**^*p* < 0.01. (**e, f**) **Western blot.** A549 cells were treated with β-Lap (7.5 µM) or/and CGA (500 µM) in the presence of 100 nM Rapa or 15 µM CQ for 2 h, and further incubated in the fresh medium with 5% FBS containing autophagy activator or inhibitor for 4 h. Total cell extracts (30 µg) were separated by 8% or 12 % SDS-PAGE and analyzed by Western blotting using primary antibodies against p-LC3(s-12), LC3, SQSTM1/P62, caspase-3, cleaved caspase-3, PARP, and cleaved PAR, p-PKAα/β/γ cat, PKA, p-mTOR (s-2448) and mTOR (**e**). β-actin was used as a loading control. The relative levels of these indicated proteins were quantified and represented as graphs (**f**).
